# Rare aggressive natural killer cell leukemia presented with bone marrow fibrosis - a diagnostic challenge

**DOI:** 10.1186/2193-1801-3-390

**Published:** 2014-07-29

**Authors:** Dina S Soliman, Ahmad Al Sabbagh, Halima El Omri, Firyal A Ibrahim, Aliaa M Amer, Ivone B Otazu

**Affiliations:** Department of Laboratory Medicine and pathology, Hamad Medical Corporation, Doha, Qatar; Department of Hematology and Medical oncology, Hamad Medical Corporation, Doha, Qatar

**Keywords:** Aggressive natural killer cell leukemia, Bone marrow fibrosis, Dysmegakaryopoiesis

## Abstract

Aggressive natural killer cell leukemia is an extraordinary rare aggressive malignant neoplasm of natural killer cells. Although its first recognition as a specific entity was approximately 20 years ago, this leukemia has not yet been satisfactorily characterized as fewer than 200 cases have been reported in the literature and up to our knowledge, this is the first case report in Qatar. Reaching a diagnosis of aggressive natural killer leukemia was a challenging experience, because in addition to being a rare entity, the relative scarcity of circulating neoplastic cells, failure to obtain an adequate aspirate sample sufficient to perform flow cytometric analysis, together with the absence of applicable method to prove NK clonality (as it lack specific clonal marker); our case had atypical confusing presentation of striking increase in bone marrow fibrosis that was misleading and complicated the case further. The bone marrow fibrosis encountered may be related to the neoplastic natural killer cells’ chemokine profile and it may raise the awareness for considering aggressive natural killer leukemia within the differential diagnosis of leukemia with heightened marrow fibrosis.

## Background

Aggressive NK- cell leukemia (ANKL) is an extraordinary rare lymphoproliferative disorder that was first described by Fernandez et al. (Fernandez et al. 1986). Imamura et al. (Imamura et al. 1990) also identified leukemia of NK-cell origin, but their findings were not published for several years. The World Health Organization Classification first recognized its aggressive clinical behavior and separated it from T-cell large granular lymphocyte leukemia, calling it Aggressive NK-cell Leukemia in 2001 (Chan et al. 2001). This entity is characterized by systemic neoplastic proliferation of NK-cells, almost always associated with Epstein-Barr virus, detected most reliably by in situ hybridization (ISH) for EBV-encoded RNA (EBER), constituting a diagnostic requisite. ANKL occurs at a relatively young age group with a median age of 42 years and strong racial predisposition as it is most prevalent among Asians (Imamura et al. 1990). The patients usually present with poor general condition, fever, and disseminated disease with rapidly progressive clinical course (Chan et al. 1997), they often die within a short time from systemic disease or complications such as multi-organ failure (Chan et al. 2008). Since the number of neoplastic cells in the peripheral blood and bone marrow can be limited compared to the usual leukemia, this disease also has been called aggressive NK-cell leukemia/lymphoma (Chan 1998). The neoplastic cells are typically CD2^+^, surface CD3-, cytoplasmic CD3ϵ^+^, CD56^+^ with germline configurations of T-cell receptor genes and expression of cytotoxic molecules (perforin, granzyme B, and TIA-1) (Chan et al. 2008; Chan et al. 2010). According to WHO (2008), NK cell neoplasms include: chronic lymphoproliferative disorder of NK cells (CLPD-NK) (a provisional entity) and the malignant subtypes including extranodal NK/T-cell lymphoma of the nasal type (ENKL) and aggressive natural killer leukemia (ANKL) (Chan et al. 2008). The first entity (CLPD-NK) is more indolent compared to fulminating prognostically adverse extranodal NK/T-cell lymphoma of the nasal type (ENKL) and aggressive natural killer leukemia (ANKL). Immunophenotypically, the latter exhibit stronger CD56-expression, larger forward scatter (FSC) and usually more CD7 negative and CD16 negative, compared to the former subtype which is CD56 dim/negative CD16 positive (Jiang et al. 2013; Trotta et al. 2005). However, all of them are associated with EBV infection (Chan et al. 2008; Nava & Jaffe 2005). CD56 is a neural cell adhesion molecule, it is a sensitive marker for NK cells, as it is expressed very early on committed NK precursors and on more than 95% of mature NK cells; its strong expression is related to the invasion and poor prognosis of neoplasms (Kwong et al. 1997).

## Case report

A 49-year-old Philipino male, not known to have any chronic illness, was admitted in April 2013 in the department of medicine with a two-month history of weight loss, epigastric pain along with loss of appetite, easy fatigability and two days history of fever.

On physical examination, the patient was pale, jaundiced with a very poor general condition (performance status 3) and no apparent skin lesions. He was febrile with temperature of 39°C, pulse 100/min and blood pressure of 100/60 mmHg.

The patient’s laboratory work-up revealed markedly raised total bilirubin at 101umol/L (n: 3.5-24) with direct fraction of 57 umol/L, low Albumin 29 g/L (n: 35–50), slightly elevated ALT 45 U/L (n: 0–40), normal total protein, AST, Alkaline Phosphatase, Creatinine, electrolytes and normal B12/folate.

Coagulation screening revealed progressively prolonged APTT with normal PT, increased D-Dimer of 4.8 mg/L (n: up to 0.55 mg/L EFU) and low protein C (46%) (n: 70-140%).

His CBC parameters showed pancytopenia with hemoglobin of 8.8 g/dl (n: 13–17), mild reticulocytosis at 5%, mildly reduced WBC 2.5 ×10/ul (n: 4–10), mild neutropenia (absolute neutrophil count of 1.8 ×10^3^/ul (n: 2–7), and marked thrombocytopenia of 20 ×10^3^/ul (n: 150–400). Serum level of lactate dehydrogenase was unexpectedly within normal 233U/L. Septic workup including blood, urine, stool and ascitic fluid culture revealed Klebsiella pneumoniae septicemia.

**Figure 1:**
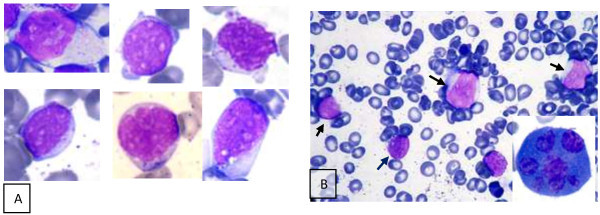
**Morphology of neoplastic cells. (A)** Composite showing examples of pleomorphic circulating leukemic cells in peripheral blood obtained from subsequent peripheral blood smears. **(B)** Bone marrow aspirate smear showing four blastoid looking cells (arrows) and dysplastic megakaryocyte with multiple widely separated nuclei) (Lower left corner).

At presentation, peripheral blood smear showed mild polychromasia, few elliptocytes and rare tear drops. The majority of circulating lymphocytes were small and mature with very rarely encountered atypical-primitive looking lymphoid cells (Figure 1A).

Viral serology disclosed heavy infection with Epstein-Barr virus with (EBV) DNA of 26,754 copies/ml by PCR with positivity for Hepatitis B surface antigen, Hepatitis B core antibody and no detectable Hepatitis B virus DNA. Hepatitis C, HIV and HTLV antibody were all negative.

Whole body CT scan revealed bilateral pleural effusion, ascites with minimal pericardial effusion, MRI of the abdomen showed hepatomegaly, splenomegaly; multiple enlarged lymph nodes noted in the inguinal, retroperitoneal, porta hepatis, mesenteric and paraaortic regions, the largest lymph nodes were at the porta hepatis.

**Figure 2:**
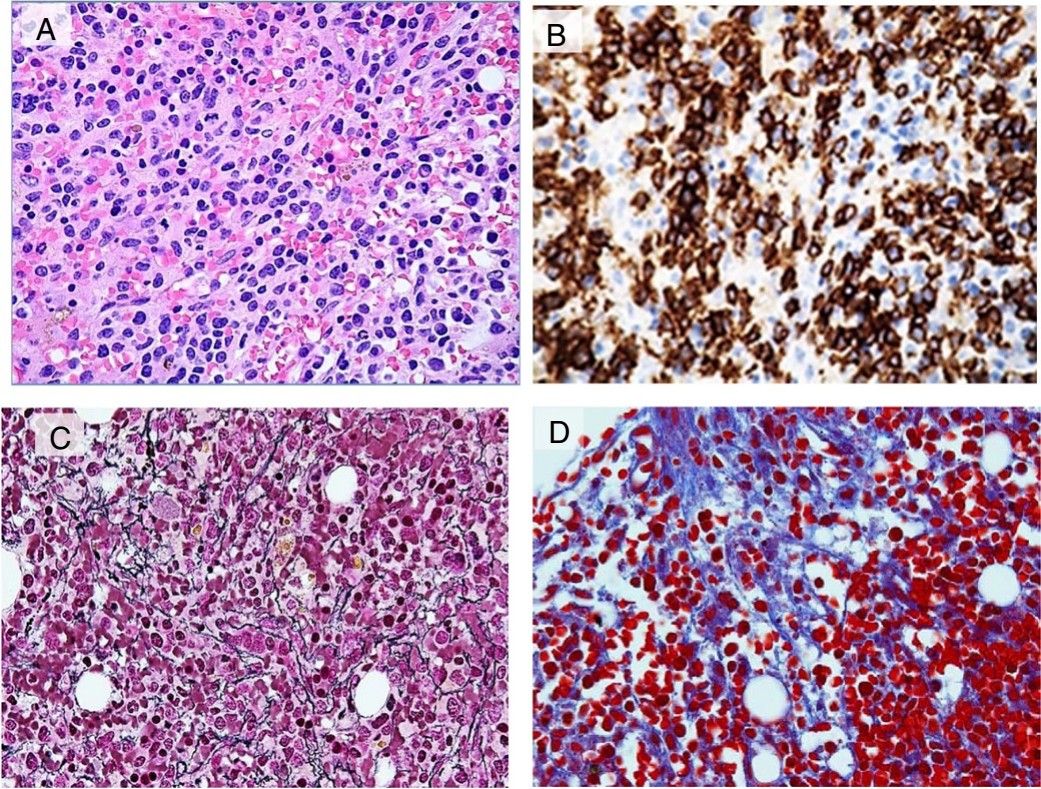
**Bone marrow biopsy findings. (A)** Biopsy section (H&E), at low magnification (20×) showing infiltration by abnormal lymphoid cells. **(B)** CD56 immunostain highlighting marked increase in NK cells (50×). **(C)** Reticulin stain highlighted an increase in reticulin fibrosis (50×), **(D)** Trichrome stain highlighting focal bundles of collagen deposition (50×).

**Figure 3:**
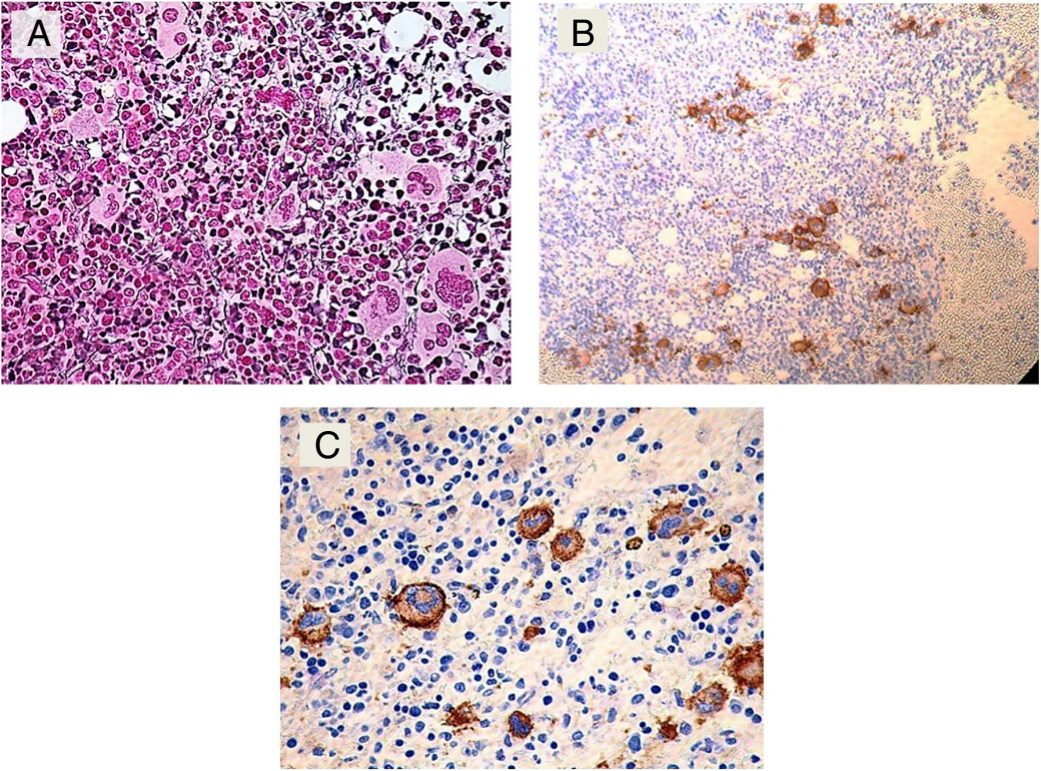
**Megakaryocytic atypia and anisocytosis. (A)** Biopsy section (reticulin stain) showing megakaryocytic atypia with small clusters, **(B)** CD61 immunostain showing megakaryocytic clustering (10×), **(C)** CD61 immunostain showing anisocytosis (50×).

Bone marrow examination was then performed; the aspirate smear was partially hemodilute, infiltrated with primitive looking lymphoid cells comprising approximately of 22%, of medium to large size with blastoid dispersed chromatin, prominent multiple small nucleoli and abundant moderately basophilic cytoplasm. Fine cytoplasmic granulations were noted in few neoplastic cells. Erythropoiesis was mildly hyperplastic with some dyserythropoietic changes, mainly in the form of megaloblastoid chromatin and nuclear irregularities encountered in about 20% of erythroid precursors. No significant dysgranulopoietic changes were noted. Examination of bone marrow biopsy revealed hypercellular marrow with few necrotic areas; that is extensively infiltrated with atypical lymphoid cells with round or irregular nuclei, rather condensed chromatin, multiple small nucleoli residing within a reactive inflammatory back ground composed of small lymphocytes, histiocytes, neutrophils, eosinophils, and polyclonal plasma cells (Figure 2A). In addition, there was striking megakaryocytic hyperplasia with some clustering, marked atypia in the form of abnormal separation of nuclear lobes with evident anisocytosis and some micromegakaryocytes (Figure 3). The dysplastic megakaryocytes were also present in the aspirate smear, however, their percentage cannot be determined due to insufficient aspiration material obtained (Figure 1B).

**Figure 4:**
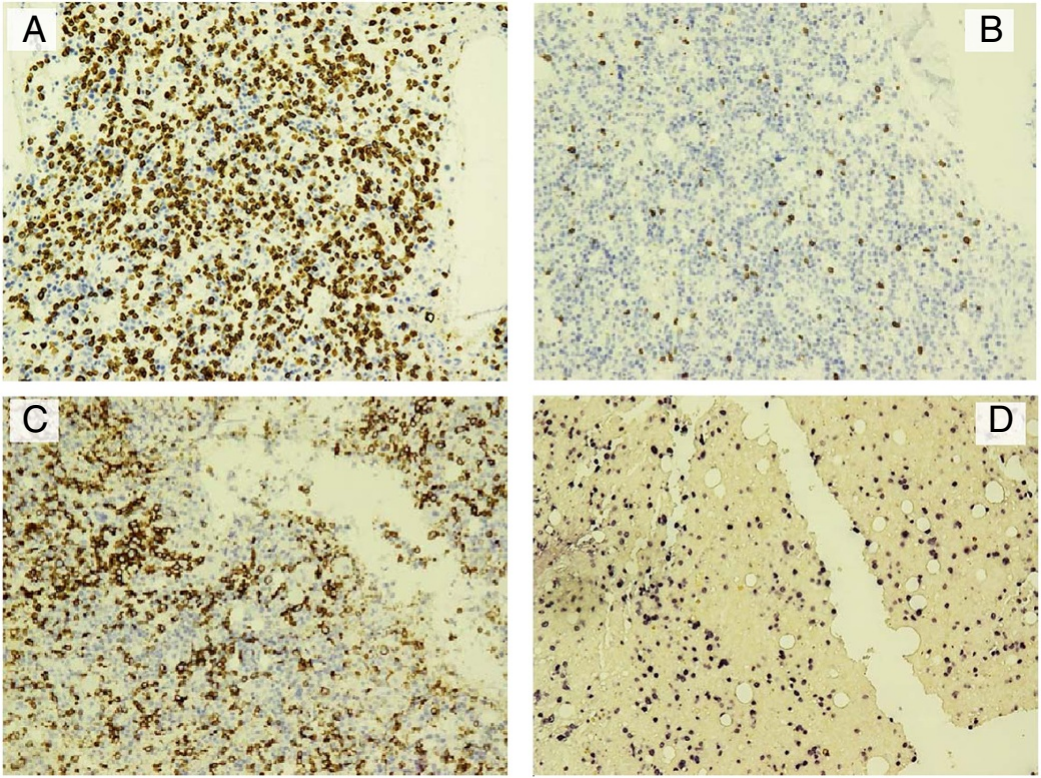
**Immunohistochemistry performed on bone marrow biopsy section (20×).** The lymphoid infiltrate is positive for CD3 (epsilon) **(A)**, negative for CD5 **(B)**, positive for CD56 **(C)** positive for EBV **(D)**.

By immunohistochemical stains, the abnormal cells were strongly positive for CD3 epsilon (CD3ϵ polyclonal, Dako, Denmark), CD56 and EBV RNA (using ISH technique) (Figure 4) and negative for CD20, CD5, CD4 & CD8 with partial weak positivity for CD7. The percentage of infiltration was approximately 60-80%.

Reticulin stain highlighted diffuse increase in reticulin fibrosis with some intersections (grade II out of III) (Figure 2C) and focal bundles of collagen deposition highlighted by Trichrome stain (Figure 2D) associated with streaming of hemopoietic cells noted in some areas of bone marrow biopsy.

**Figure 5:**
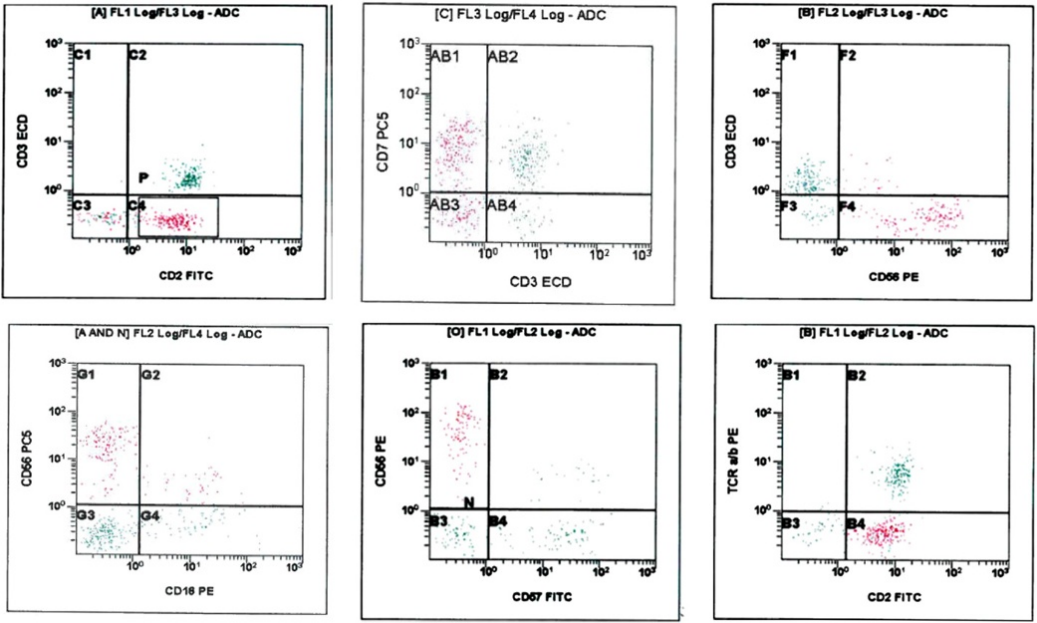
**Flow cytometry performed on ascitic fluid.** The population of interest expresses CD2, CD7 and CD56, and lacks surface CD3, CD16, CD57 and TCR alpha-beta. Abbreviations ECD, phycoerythrin-Texas Red; FTIC, fluorescein isothiocyanate; PE, phycoerythrin.

Cervical lymph node biopsy was inconclusive as it revealed reactive changes with picture suggestive of dermatopathic lymphadenopathy and did not raise the suspicion of involvement by NK malignancy. Flow cytometry performed on lymph node revealed moderate increase in NK cells. Flow cytometry was then performed on ascitic fluid using antibody panel: CD45, CD2, sCD3, CD5, CD7, CD4, CD8, CD56, CD57, CD16, TCR alpha-beta and TCR gamma-delta, it revealed approximately 20% NK-cells expressing CD2, CD7 & CD56, they were negative for CD3 (surface), CD4, CD5, CD8, CD57, TCR alpha-beta and TCR gamma-delta (Figure 5).

In order to R/O nasal or sinus involvement, CT and MRI head were carried out and it showed clear nasal passages and paranasal sinuses with no mass lesions.

**Figure 6:**
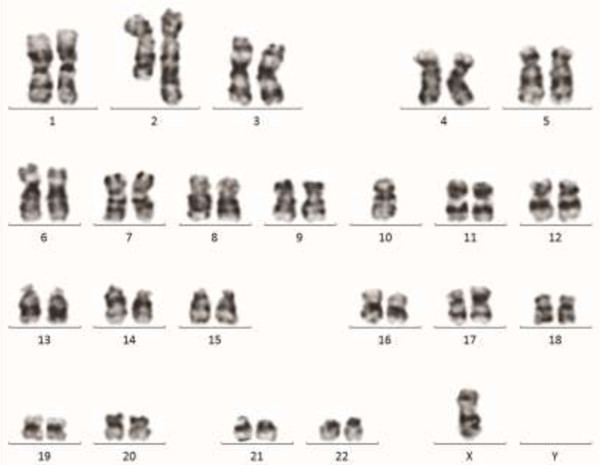
**Karyotype by conventional cytogenetics.** ISCN:44,X,-Y, der(2) t(2;10) (q37;q11.2), der(17) (p?11.2) (Zhang et al. 2012). A minimum of 20 metaphases were karyotyped according to the International System for Human Cytogenetic Nomenclature (Shaffer et al. 2009), image capturing, processing and data were carried out using microscope Zeiss-AX10 scope.A1 with IKAROS imaging system (Metasystems, Germany).

Conventional Cytogenetic analysis using GTG-banding was performed on unstimulated 24 and 48 hours culture of bone marrow aspirate according to standard procedures; it revealed an abnormal clone with complex karyotype (≥3 aberrations) consisting of aneuploidy cells with 44, X, (missing the sex chromosome Y), derivative chromosome 17, derivative chromosome 2 that resulted from reciprocal translocation between the chromosomes 2 and 10 at bands q37 and q11.2, respectively, besides an additional material of unknown origin attached at band p?11.2 in 15 cells (Figure 6). Molecular Cytogenetics (FISH) analysis was performed using LSI TP53 SO/CEPSG which allows the distinction of the chromosomes 17 (p53 gene) and MLL gene (11q23) LSI MLL Dual color, Break Apart Rearrangement probe (Abbott, molecular, Vysis, Des Plaines, IL, USA). A total of 300 interphase cells and at least seven abnormal metaphase cells each were analyzed by FISH. Deletion of the p53 gene was confirmed by FISH in 11% of the nuclei cells analyzed. Interphase nuclei cells showed normal hybridization pattern when MLL gene was analyzed. During the patient’s stay in the hospital, his general condition was deteriorated with increase of the tumor burden, aggravation of ascites, hepatosplenomegally, and lymphadenopathy along with progressive pancytopenia and increase in the percentage of the abnormal lymphoid cells in the peripheral smear (Figure 1A).

Since admission, the patient was started on supportive measures including albumin, antibiotics and antiviral (entecavir 0.5 mg). As the patient developed significant anemia and thrombocytopenia, he received multiple packed RBC and platelets transfusion. He developed an autoimmune hemolytic anemia with strong positivity for direct coomb’s test (DAT), (++) IgG and reticulocytosis.

In view of the pathologic findings and the clinical picture, a diagnosis of aggressive NK cell leukemia was made. After confirmation of the diagnosis, SMILE protocol (dexamethasone, methotrexate, ifosfamide, l-asparaginase, and etoposide) was planned but unfortunately, the patient’s response could not be monitored as he travelled back to his home country before initiation of therapy.

## Discussion

Aggressive natural killer cell leukemia (ANKL) is a rare aggressive malignant neoplasm of natural killer cells (NK), only recently recognized, with a median survival of less than 2 months (Sokol & Loughran 2006) and a strong association with the Epstein-Barr virus. Although the first recognition of ANKL as a specific entity was approximately 20 years ago, this leukemia has not yet been satisfactorily characterized as fewer than 200 cases have been reported in the literature. In flow cytometry immunophenotyping, the definition of NK cell is the lymphocyte on which CD3 is negative, CD16 and/or CD56 are positive. Abnormal NK cells are similar to normal NK cells in immunophenotype, they express CD2, CD7, CD11c, CD16, and CD56. CD3 and CD4 are not expressed in all NK cells (Jiang et al. 2013).

The lack of a uniquely rearranged antigen receptor gene in natural killer (NK) cells that can be used to detect clonal cellular expansion has served as a stumbling block in establishing the presence of NK cell neoplasm. At present, the identification of abnormal NK cells mostly relies on the pathological morphology and immunohistochemistry.

Fewer than 100 cases of ANKL were reported in the English- language literature till 2010 (Gogia et al. 2010) involving Japanese, Korean, Chinese and Caucasians patients. 20 more cases were studied in a Chinese study published in 2012 (Zhang et al. 2012), 3 more Japanese cases were reported in 2012 (Okuno et al. 2012), one case was reported in Danish study (Boysen et al. 2011) and additional 43 cases of ANKL were involved in another large Chinese study conducted in the time period between 2006–2012 and published in 2013 (Jiang et al. 2013). Up to our knowledge this is the first case report in Qatar.

In our case the diagnosis was challenging and somewhat delayed; this was especially caused by lack of sufficient BM aspiration enough to perform immunophenotyping by flow cytometry, in addition to the rarity of circulating neoplastic cells. Furthermore, morphologically, the neoplastic cells had a primitive blastoid appearance with no specific distinguishing features. Unlike NK lymphomas, with a more mature phenotype, no clear cytoplasmic granules were detected by light microscopic examination. Furthermore, the CD3-positive lymphoid infiltrate of the bone marrow biopsy showed interstitial pattern that can be conceived as an inflammatory infiltrate particularly in the presence of active EBV viral infection. The striking finding was the presence of increased reticulin fibrosis with rather megakaryocytic atypia, a finding that added more confusion and complicated the case further.

Disorders associated with increased BM fibrosis including primary myelofibrosis were initially considered in the differential diagnosis especially in view of pancytopenia, hepatosplenomegally, leucoerythroblastic picture, and increased blastoid looking cells.

This possibility soon vanished after examination of the immunostains which highlighted massive interstitial infiltration with CD3 positive cells with no increase in CD34 positivity by immunohistochemistry (<1%).

The CD3 used in immunohistochemistry is CD3ϵ that is specific for cytoplasmic epsilon chain found in both T cells and NK cells. The next question was are these true T cells or NK cells with cytoplasmic CD3 expression?, the answer came shortly after the second immunohistochemical panel (which included T-cells and NK-cells’ more specific markers), favoring NK cell origin with negativity for CD5, CD4, CD8, CD57, strong positivity for CD56+, granzyme B and weak positivity for CD7.

The whole picture was then consistent with NK-cell neoplasm, however, because of the lack of a hallmark diagnostic marker, the distinction between this groups of diseases can be confusing, as they share some pathologic features and similar immunophenotypes. CLPD-NK was initially omitted as we did not have persistently increased NK cells (≥2 × 109/L), a criterion required by the WHO for this diagnosis. It has been suggested that aggressive NK-cell leukemia may represent the leukaemic phase of extranodal NK-T-cell lymphoma, however, there are some distinctive features for the former: younger median age, high frequency of hepatosplenic and BM involvement, low frequency of skin involvement and disseminated disease(Zhang et al. 2012).

Demonstration of strong EBV positivity by Epstein-Barr encoding region (EBER) in situ hybridization (EBER-ISH) was another supporting evidence, it is considered a diagnostic requisite as more than 90% of cases harbor EBV, which occurs in clonal episomal form (Au et al. 2004). EBV-negative cases should be diagnosed as peripheral T-cell lymphoma, not otherwise specified (Chan et al. 2008). Some studies showed that plasma EBV DNA was correlated with tumor load, with high EBV DNA correlating negatively with survivals (Au et al. 2004). Serial EBV DNA monitoring is useful for assessing response, detecting recurrence (Au et al. 2004) and may be useful in risk stratification for HSCT (Eric & Yok-Lam 2013).

There is controversy about CD16 expression in cases of ANKL, some studies reported relatively high expression rate of CD16 up to 75% (Suzuki et al. 2004) and 50% (Ryder et al. 2007), and others revealed absence (Gogia et al. 2010) or very low expression rate of CD16 (Jiang et al. 2013). Anyhow, our case showed no significant expression of CD16 by flow cytometry performed on ascitic fluid.

Unlike previous reports which did not report lymphadenopathy (Zhang et al. 2012) or reported it at a low incidence (Jiang et al. 2013; Ryder et al. 2007; Nakashima et al. 2005), our case had generalized lymphadenopathy. However, both the lymph node biopsy and flow cytometry on cervical lymph node failed to characterize an abnormal phenotype required to confirm LN involvement with neoplastic process.

There are no specific recurrent cytogenetic abnormalities have been identified in ANKL, a variety of clonal cytogenetic abnormalities have been reported such as del (6) (q21q25) and 11q deletion (Zhang et al. 2012; Nakashima et al. 2005). Our case harbored a complex karyotype with P53 deletion and novel t (2; 10) (q37; q11.2), the latter has not been previously reported in NK-cell malignancies.

The precise role of the p53 point mutation in ANKL leukemogenesis remains to be clarified, as there are no reports correlating p53 mutation in ANKL leukemia with disease progression (M Yagita et al. 2000).

Some previous reports had largely relied on flow cytometry to characterize the immunophenotype of the neoplastic cells in cases of ANKL and found it helpful in the differential diagnosis of NK cell neoplasms; based on the immunophenotypic differences among ANKL, CLPD-NK and normal NK cells (Jiang et al. 2013; Ryder et al. 2007; Ruskova et al. 2004). This was however, disagreed by Hongyu Z et al. (Zhang et al. 2012) who had concluded that flow cytometry was of limited value, as only two out of 20 ANKL patients had aspirate smears with sufficient malignant cells enough to confirm the phenotype by flow cytometry. In our case, bone marrow sample (although repeated) was consistently suboptimal for flowcytometric analysis. It is however, worth noting that relatively late in the course of the disease, NK cell origin was further supported by flow cytometric analysis performed on ascitic fluid. Jiang et al. (Jiang et al. 2013), found that 6 out of 11 patients were diagnosed in the second or third bone marrow biopsy when the disease had already progressed. In our case, we believe that the cause behind suboptimal aspirate sample obtained was most likely related to extensive bone marrow fibrosis encountered. However, this finding was not previously reported as a possible cause for difficult sampling. Few previous studies reported bone marrow stromal damage including focal reticulin fibrosis (Ryder et al. 2007). The striking bone marrow fibrosis noted in our case is far exceeding what usually seen in other cases of acute leukemia and up to our knowledge it is not previously reported in this disease entity. The presence of an associated contributing factor, although unlikely cannot be entirely ruled out.

While hemophagocytosis and dyserythropoiesis appear to be the most commonly reported morphologic abnormalities in the bone marrow (Chan et al. 1997; Ryder et al. 2007). Prominent dysmegakaryopoiesis was rarely reported in the literature (Ruskova et al. 2004).

Our case was found to be a chronic carrier for hepatitis B, since hepatitis B virus carrier rates are high in populations at risk of NK/T-cell lymphoma; it is recommended that hepatitis B virus testing should be routine and antiviral prophylaxis administered to carriers during chemotherapy (Liang et al. 1999). Philipp A. Lang, et al (Lang et al. 2012) has identified a negative regulatory role for NK cells during both acute and chronic virus infection which may be linked to reduced clearance of the virus and predispose to chronic carrier status.

One reason for the fulminant clinical course and adverse outcome in ANKL is leukemic infiltration into multiple organs through a chemokine mediated cell trafficking (Makishima et al. 2007). Among various chemokine receptors tested, CXCR1 and CCR5 were simultaneously expressed on ANKL cells and the leukaemic cells showed enhanced chemotaxis towards the chemokines of these receptors (Kato et al. 1998). Further, the serum levels of the corresponding chemokines interleukin IL-8, RANTES, macrophage inflammatory protein (MIP)-1α and MIP-1β, soluble FasL are significantly elevated in ANKL patients (Kato et al. 1998). In addition, ANKL cells were found to express high levels of these chemokines detected by intracellular staining and RT-PCR (Makishima et al. 2007). Interaction between these chemokines produced by hepatocytes and chemokine receptors on leukaemic cells may play an important role in the transmigration of tumor cells to hepatocytes (Makishima et al. 2007) that induces inflammatory environment and subsequent liver fibrosis. Additionally, chemokines such as MIP-1, MCP-1, and IL-8 have also been hypothesized to play key roles in the recruitment of leucocytes to fibrotic lungs and promotion of the pro-fibrotic environment therein (Palmqvist et al. 2007). In our opinion, a similar mechanism of chemokines induced marrow fibrosis might be implicated in the pathogenesis of accentuated bone marrow noted.

In conclusion, early diagnosis of aggressive NK leukemia remains very difficult, as compared with T or B cells neoplasms; the absence of specific receptor gene rearrangement prohibits the recognition and characterization of NK-lineage malignancies. Although, flow cytometry serves a reliable tool in differentiation between T cell and NK cell phenotype, it is often difficult to obtain a satisfactory marrow sample sufficient to perform the analysis that permits early diagnosis. In agreement with what have been reported previously (Zhang et al. 2012). We found that bone marrow biopsy complemented by immunohistochemistry and EBER detection provide the most reliable approach for the diagnosis. CD3ϵ and CD56 are the most important antigens for recognition of NK cells by immunohistochemistry. It is even suggested that if only paraffin material is available, the diagnosis of ANKL can still be made, provided that CD56, EBER, and cytotoxic molecules are positive (Chan et al. 2008).

Lymph nodes enlargement is not reported frequently in this entity, moreover, it appears that the detection of abnormal NK infiltrate in the lymph node is difficult to characterize and this may raise a question regarding the actual involvement of the lymph nodes by the neoplastic cells.

The extensive bone marrow fibrosis encountered in this case may be related to the neoplastic NK cells’ chemokine profile and it should raise the awareness for considering ANKL within the differential diagnosis of leukemia with heightened marrow fibrosis.

As extensive bone marrow fibrosis is not previously reported in cases of ANKL, more studies are required to assess the frequency of this finding and to expose any possible role that might be played by fibrosis in the pathogenesis of ANKL and its destructive behavior.

Being an extraordinarily rare entity, as more cases of aggressive NK leukemia/lymphomas are being reported, our understanding of the clinical and pathological spectrum of this fatal malignancy is evolving.

## Abbreviations

ANKL: Aggressive natural killer cell leukemia
CLPD-NK: Chronic lymphoproliferative disorder of NK cells
ENKL: Extranodal NK/T-cell lymphoma of the nasal type.
